# Repurposing Masitinib Mesylate as a Novel FOXM1 Inhibitor for the Treatment of Aggressive Solid Tumors: Preclinical Validation in Human Breast and Oral Cancer Cells and Organotypic Tumor Slice Culture

**DOI:** 10.1021/acsomega.6c02787

**Published:** 2026-06-29

**Authors:** Rajat Gupta, Jai Singh, Shruti Dayanand Shetty, Naveena A. N. Kumar, Adarsh Kudva, Sandeep Kumar Srivastava, Sanjiban Chakrabarty

**Affiliations:** † Department of Public Health Genomics, 539679 Manipal School of Life Sciences, Manipal Academy of Higher Education, Manipal 576104, India; ‡ Structural Biology & Bioinformatics Laboratory, Department of Biosciences, 385092 Manipal University Jaipur, Jaipur 303007, Rajasthan, India; § Department of Biochemistry, University of Allahabad, Prayagraj 211002, Uttar Pradesh, India; ∥ Department of Surgical Oncology, Manipal Comprehensive Cancer Care Centre, 29224 Kasturba Medical College, Manipal Academy of Higher Education, Manipal 576104, Karnataka, India; ⊥ Department of Oral and Maxillofacial Surgery, 29228 Manipal College of Dental, Manipal Academy of Higher Education, Manipal 576104, Karnataka, India

## Abstract

The Forkhead box M1 (FOXM1) transcription factor, recognized as a prominent oncogene, facilitates cell cycle progression and mitotic fidelity in aggressive cancers. Overexpression of FOXM1 is correlated with uncontrolled cell proliferation, metastasis, and poor prognosis. Despite its pivotal role in promoting genomic instability, cancer stemness, and metastasis, FOXM1 remains a clinically undruggable target. In this study, we identified masitinib mesylate, a small-molecule inhibitor known for its multitargeted tyrosine kinase inhibition, targeting c-Kit, PDGFRα, and PDGFRβ, as well as Lyn, which demonstrates anticancer activity in preclinical models. Masitinib mesylate emerges as a potent, novel first-in-class small-molecule inhibitor of FOXM1. *In silico* prediction using pharmacophore-based virtual screening revealed that masitinib mesylate binds with high affinity to the FOXM1 DNA-binding domain (DBD) through hydrogen bonding with residues at His287 and Leu291, as well as a salt bridge with Asp293, effectively stabilizing the FOXM1-DNA interface and perturbing its activity. *In vitro* validation in the triple-negative breast cancer cell line MDA-MB-231 and the highly aggressive oral cancer cell line HSC-3 demonstrated that masitinib mesylate significantly reduces FOXM1 protein expression, thereby contributing to reduced cancer cell proliferation, stemness, and migration. Importantly, we validated these findings using *ex vivo* human primary tumor slice cultures from patients with breast cancer and oral squamous cell cancer. Treatment with masitinib mesylate significantly induced apoptosis within the native tumor microenvironment, confirming its efficacy in heterogeneous, patient-derived tissues. Our findings establish a novel mechanistic link between masitinib mesylate and FOXM1 inhibition by DNA binding, providing a compelling rationale for drug repurposing, offering a targeted therapeutic strategy to disrupt the FOXM1-driven regulatory network in aggressive, therapy-resistant cancers. *In silico* analyses predict a potential interaction of masitinib with the FOXM1 DNA-binding domain (DBD); however, further experimental validation is required to confirm direct binding.

## Graphical abstract

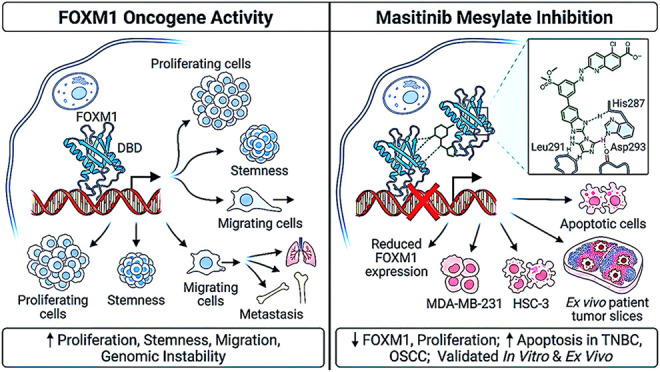

## 1 Introduction

The Forkhead box M1 (FOXM1) transcription factor serves as a crucial regulator of cellular proliferation, cell cycle progression, DNA damage repair, and the evasion of apoptosis, thereby playing a central role in tumor development and progression across various human cancers. FOXM1 drives key cell cycle transitions (G1/S and G2/M) by transcriptionally activating genes such as cyclin B1, PLK1, and CENPF, and is frequently overexpressed in aggressive malignancies, including triple-negative breast cancer (TNBC) and oral squamous cell carcinoma, which correlates with poor prognosis, metastasis, and therapy resistance. Its oncogenic role extends through promoting genomic instability, cancer stemness, metabolic reprogramming, angiogenesis, invasion, and multidrug resistance.^1–4^ Masitinib is an orally bioavailable, potent, and selective tyrosine kinase inhibitor that targets c-Kit, PDGFRα/β, and Lyn kinases and has demonstrated antitumor activity in various preclinical cancer models and clinical trials.^5^ It has shown efficacy in inhibiting tumor cell migration and proliferation, modulating the tumor microenvironment, and overcoming drug resistance mechanisms.^5–7^

Head and neck squamous cell carcinoma (HNSCC) is characterized by one of the most lethal and frequently occurring malignancies known to humans. HNSCC patients have a high degree of tumor recurrence and poor progression-free survival.^8^ The therapeutic approaches for treating locoregionally confined HNSCC are radiation, surgical resection, and chemotherapy. For advanced-stage patients, definitive therapy with chemoradiotherapy rather than a surgical regimen is given to early stage patients with HNSCC.^9^ However, even after advanced treatment, patients relapse. The use of multiple-drug chemotherapy before definitive chemoradiotherapy in tumors has been studied extensively. Nevertheless, there was no improvement in overall survival compared with radiation and high-dose cisplatin. Breast cancer is the most common female cancer worldwide, and although many patients are cured, approximately 20% develop metastasized disease, which is usually incurable.^10^ Among the different breast cancer subtypes, triple-negative breast cancer (TNBC) patients, who lack expression of ER, PR, or human EGF receptor 2 (HER2) in their tumors, have the worst outcome with limited treatment options.^11^ Furthermore, in the case of recurrence or metastatic HNSCC and TNBC, few patients receive reirradiation or mastectomy. However, the majority of the patients with advanced disease are considered for palliative therapy and pain management to maintain quality of life and end-of-life support.

Previously, several studies have reported FOXM1 inhibitors that modulate FOXM1 activity and expression levels, either directly or indirectly. Among them, FDI-6,^12,13^ XST-119,^14^ and RCM-1 inhibit FOXM1 activity by interacting with key residues Asn283, Arg286, and His28 within the DNA-binding domain (DBD) and with DNA bases, thereby preventing its interaction with target genes. The other known FOXM1 inhibitors, such as honokiol, thiostrepton, and sinomycin A, inhibit the expression or activity of upstream kinases and coactivators which regulate FOXM1 transcriptional activity.^15,16^ Due to off-target effects, the majority of these FOXM1 inhibitors have limited or no success in clinical trials.^17,18^

Given that FOXM1 is a master regulator of aggressive tumor behavior and that masitinib targets tyrosine kinase pathways essential for cancer cell survival and migration, exploring the effects of masitinib on FOXM1-driven cancers is warranted. The present study examined the anticancer efficacy of masitinib in two FOXM1-overexpressing human cancer cell lines: MDA-MB-231 (triple-negative breast cancer) and HSC-3 (oral squamous cell carcinoma), followed by treatment in patient-derived organotypic tumor slice culture. We evaluated the dose-dependent cytotoxic effects of masitinib by determining IC_50_ values using MTT assays, assessed its impact on cell proliferation through EdU incorporation microscopy, and analyzed alterations in FOXM1 protein expression *via* Western blotting. This integrative approach elucidates both the functional and molecular consequences of masitinib treatment in diverse cancer models with elevated FOXM1 activity, providing novel insights into the potential repurposing of masitinib as a modulator of FOXM1 signaling in aggressive breast and head and neck cancers.

## 2 Materials and Methods

### 2.1 Protein Data Acquisition and Identification of Protein–DNA Residues

The crystal structure of FOXM1 with bound DNA (PDB ID-3G73) at a resolution of 2.21 Å is available in the PDB database^19^ was used in *in silico* docking calculations. All the interactions between protein–DNA complexes were analyzed, and accordingly, all the heteroatoms, water molecules, and bound ligand atoms were removed from the PDB file to prepare the target protein for docking runs *via* the PyMol tool.^20^

### 2.2 Pharmacophore-Based Virtual Screening

Pharmacophore-based screening is a vital step in searching for leads within large libraries, using defined parameters to improve accuracy against a specific target in computational drug development. Pharmacophore models involve a set of shared steric and electronic characteristics crucial for optimal interactions with a given target, which may initiate or inhibit its biological response.^21^ Diverse interaction features, including hydrogen bond donors (HBDs), hydrogen bond acceptors (HBAs), and aromatic and cationic characteristics, are employed to define interaction patterns,^22^ which are then utilized to evaluate the similarity among small molecules in a library. Pharmit, an online server, was used to screen small compound libraries based on pharmacophore features.^23^ We screened two small compound libraries, an FDA-approved anticancer library and a natural product library from the Selleckem database, using RCM-1 as a reference compound.^24^ The compounds were further screened using a built-in molecular weight filter (MW ≤ 500 g/mol).

### 2.3 Receptor and Ligand Preparation for Molecular Docking

Molecular docking is a computational technique used to predict the preferred orientation and binding mode of a small-molecule ligand within the active site of a target macromolecule by employing algorithmic simulation to predict its binding affinity and most energetically favorable binding mode in the ligand–receptor complex.^25,26^ To understand the binding interaction of the screened compound with the FOXM1 protein, molecular docking studies were conducted *via* AutoDock Tools 1.5.6 (ADT).^27^ The graphical user interface of AutoDock Tools was utilized to convert PDB files to PDBQT files for both the ligands and the receptor, incorporating polar hydrogen atoms, Kollman charges, and solvation parameters. A grid box of size 40 × 40 × 40 points along the *x*, *y*, and *z* directions with a 0.375 Å grid spacing centered with dimensions along *x*, *y*, and *z* of 13.10, 25.04, and 9.12 Å, respectively, was generated *via* ADT to run the AutoDock Genetic Algorithm program. For each compound, ten docking conformations were generated and ranked on the basis of their docking energy, and hits with a docking score greater than the reference inhibitor were selected.

### 2.4 ADMET Profiling

ADMET (absorption, distribution, metabolism, excretion, and toxicity) plays a crucial role in determining a drug’s pharmacokinetics and pharmacodynamics. These properties were assessed *via* the SwissADME Web site. ADMET features encompassing the “topological polar surface area, AMES toxicity, and gastrointestinal absorption (GIA)” of selected ligands were predicted and examined.^28^ To calculate the toxicological properties, SMILES notations for each hit were submitted to the ADMETlab 3.0 Web site.^29^ The compounds associated with all the pharmacokinetic parameters were selected for further validation using molecular dynamics simulations.

### 2.5 Molecular Dynamics Simulations and Trajectory Analysis

The screened compounds in complex with FOXM1 and the FOXM1-RCM-1 complex as a control were subjected to molecular dynamics simulations for 200 ns to understand the conformational dynamics and to define the interaction pattern of these docked complexes by using GROMACS 2023.2.^30^ The CHARMM36 all-atom force field was applied for all runs.^31^ The system was solvated in a rectangular box filled with TIP3 water molecules, with a 10 Å distance from the solute to the edge of the box. Na^+^ and Cl^–^ ions at a concentration of 0.15 M were used to neutralize the system *via* the Monte Carlo method. The solvated system was minimized in 5000 steps *via* the steep descent algorithm, and system equilibration was performed *via* 125 ps NVT ensembles at 310 K and 1 atm pressure *via* the Nose–Hoover scheme with a 1.0 ps and isotropic Parrinello Rahman barostat, which has a compressibility of 4.5 × 10^–5^ bar −1 and a coupling time constant of 5.0 ps. We used the LINCS algorithm to constrain the covalent bonds containing hydrogen atoms.^32^ The trajectories were assessed *via* the tools within the GROMACS suite: *gmxtrjconv, gmxrms, gmxrmsf, gmxgyrate*, and *gmxsasa.* All trajectory analyses were conducted with VMD 1.9.3 and the XMGRACE software package.^33,34^

### 2.6 Cell Culture

MDA-MB-231 human breast adenocarcinoma cells (triple-negative breast cancer line) and HSC-3 human oral squamous cell carcinoma cells were utilized in this study. Both cell lines were maintained in Dulbecco’s modified Eagle’s medium (DMEM) supplemented with 10% fetal bovine serum (FBS), 100 U/mL penicillin, and 100 μg/mL streptomycin. The human keratinocyte cell line HaCaT and the human embryonic kidney cell line HEK293 were kindly provided by Dr. Shama Prasada Kabekkodu. The cells were cultured in a humidified atmosphere containing 5% CO_2_ at 37 °C. The cells were passaged when they reached 80–90% confluence, and trypsin-EDTA was used for detachment. Cells were routinely tested for mycoplasma contamination.

### 2.7 Cell Viability Assay

To evaluate the cytotoxic effects of masitinib mesylate (Selleckchem: E0814), HSC-3, MDA-MB-231, HaCaT and HEK293 cells were treated with various concentrations of the compound. The cells were seeded at 5 × 10^3^ cells per well in 96-well plates and allowed to attach overnight. Following treatment with serial dilutions of masitinib for 72 h, the cells were incubated with MTT solution (1 mg/mL) for 4 h at 37 °C. The resulting formazan crystals were dissolved in DMSO, and the absorbance was measured at 570 nm *via* a microplate reader. Cell viability was calculated as a percentage relative to untreated controls.

### 2.8 Cell Proliferation Assay

The effects of FOXM1 inhibitors on cell proliferation were assessed *via* the trypan blue dye exclusion method. MDA-MB-231 and HSC-3 cells were seeded in 6-well plates and treated with selected inhibitors for 24, 48, or 72 h. The cells were harvested, stained with trypan blue, and counted using a hemocytometer to determine the percentage of viable cells relative to the control groups.

### 2.9 Colony Formation Assay

To evaluate the long-term antiproliferative effects of the inhibitors, a colony-forming assay was performed. Breast cancer cell line (MDA-MB-231) and head and neck cancer cell line (HSC-3) were treated with selected inhibitors and allowed to grow for 14 days. Colonies were then stained with crystal violet and counted to assess the compounds’ inhibitory effects on cancer cell proliferation. This assay further validated the compounds’ effectiveness in suppressing tumor cell growth.

### 2.10 Western Blot Analysis

Western blot analysis was performed to determine FOXM1 expression levels in cancer cells treated with the specified agent. Total protein was extracted from treated and control cells *via* RIPA buffer supplemented with protease and phosphatase inhibitors. Protein concentrations were determined *via* the Bradford assay. Protein lysates were subjected to SDS–PAGE, followed by transfer onto nitrocellulose membranes. The membranes were probed with specific antibodies against FOXM1 (1:2000) (Cell Signaling Technology, United States), anti-Cyclin B1 (CCNB1) (1:5000) (Cloud-Clone Corp., Cat. No. PAD264Hu01, rabbit polyclonal, ), with β-actin (1:5000) as a loading control and a secondary anti-rabbit (1:10000) (Cell Signaling Technology, United States) antibody. The relative band intensities were quantified to assess the impact of the inhibitors on FOXM1 signaling pathways.

### 2.11 Clinical Study

Breast and HNSCC tumor tissue was used for research purposes, in accordance with the code of proper secondary use of human tissue, as per the ICMR ethical guidelines for biomedical research, and was approved by the Kasturba Medical College and Kasturba Hospital Institutional Ethics Committee (IEC1:1342023 and IEC1:451/2023). Specimens were anonymized such that patient information could not be traced by the research personnel. Fresh tumor tissue was obtained from head and neck cancer (HNSCC) and breast cancer patients undergoing surgery at the Kasturba Medical College (KMC), Manipal, India, after obtaining written informed consent. Tumor samples were stored at 4 °C and transported to the laboratory in specialized culture medium within 1 h of surgical resection.

### 2.12 Organotypic Tumor Slice Culture

Tumor tissue preparation methods were adapted as described previously.^35^ Tumor specimens were subjected to automated tissue slicing under semisterile conditions. Automated slicing was performed using a Leica VT 1200S Vibratome with a slice thickness of 300 μm, a vibration amplitude of 2.0 mm, and a slicing speed of 0.6 mm/s. Unless stated otherwise, tumor slices were cultured in a medium consisting of advanced DMEM/F-12 supplemented with 1% penicillin-streptomycin and freshly added 20 ng/mL epidermal growth factor (EGF; Sigma-Aldrich, cat. no SRP3027) within 2 h after surgical resection. Culturing was performed at 37 °C, 5% CO_2_, and atmospheric oxygen levels. Culture dishes were subjected to continuous rotation at 60 rpm using a mini orbital shaker placed in the incubator, and masitinib treatment was initiated immediately after sample processing at the indicated concentrations in the culture media. Proliferating cells were labeled using 30 μmol/L EdU (Invitrogen, Waltham, MA, USA, cat. no. C10086) 2 h before fixation. Post treatment, tumor slices were fixed in 10% neutral buffered formalin for at least 24 h at room temperature. Subsequently, tumor slices were embedded in paraffin, and 5 μm sections were generated for microscopy analysis.

### 2.13 Tumor Tissue Slice Staining and Analysis

Histological tumor architecture was examined by hematoxylin and eosin (H&E) staining. Immunostaining was performed as described previously.^35^ Cell proliferation was assessed *via* an EdU (5-ethynyl-2′-deoxyuridine) incorporation assay. EdU is a thymidine analogue that is incorporated into newly synthesized DNA during the S phase of the cell cycle. Following EdU detection, the cell nuclei were counterstained with DAPI (10 μg/mL) for 10 min. The cells were washed three times with PBS and mounted with antifade mounting medium. EdU-positive cells were visualized and captured *via* a ZEISS Axioscope 5 fluorescence microscope at 400× magnification. At least 10 random fields per condition were analyzed, and the percentage of EdU-positive cells was calculated by counting the number of nuclei relative to the total number of DAPI-stained nuclei, and distributional changes were assessed using quartile-based analysis. The TUNEL (Roche Life Sciences) assay was performed to assess cell viability according to the manufacturer’s protocol.

### 2.14 Quantitative Image-Based Cytometry (QIBC)

Cells were pulse-labeled with EdU prior to fixation and DAPI staining. Images were acquired using ZEISS Axioscope 5 fluorescence microscope with a 400× magnification. QIBC analysis was performed using CellProfiler v4.2.8. Nuclei were segmented from DAPI images, and EdU incorporation was quantified within individual nuclei. DNA content was determined from integrated DAPI intensity, and S-phase cells were identified by EdU intensity. G1 and G2/M populations were separated based on DAPI intensity distribution.^36^ Scatter plots and cell-cycle quantification were generated in R.

### 2.15 Statistical Analysis

All experiments were performed in triplicate and repeated at least three times independently. The data are presented as the means ± standard errors of the means (SEMs). Statistical comparisons were performed *via* Student’s *t* test for two-group comparisons or one-way ANOVA. P values <0.05 were considered statistically significant.

## 3 Results

### 3.1 FOXM1-DNA Interaction Residue Analysis

The identification of interacting residues between FOXM1-DBD and DNA is crucial for screening compounds that bind to the FOXM1-DNA interface. We analyzed the FOXM1-DNA complex (PDB ID 3G73) using the UCSF Chimaera visualization system (Figure 1A). A comprehensive review of the literature regarding the FOXM1-DNA-binding domain (DBD) was conducted to identify the binding site residues. Asn-283, Arg-286, and His-287 are the primary residues that form hydrogen bonds with the major groove of the DNA backbone^37^ (Figure 1B). His287 forms water-mediated direct and indirect H-bonds with the DNA backbone, and Asn283 forms two hydrogen bonds with DNA along with Arg286.^25,26^ We selected these residues for grid generation and interaction pattern analysis during the screening process to understand their significance in the FOXM1 protein.

**Figure 1:**
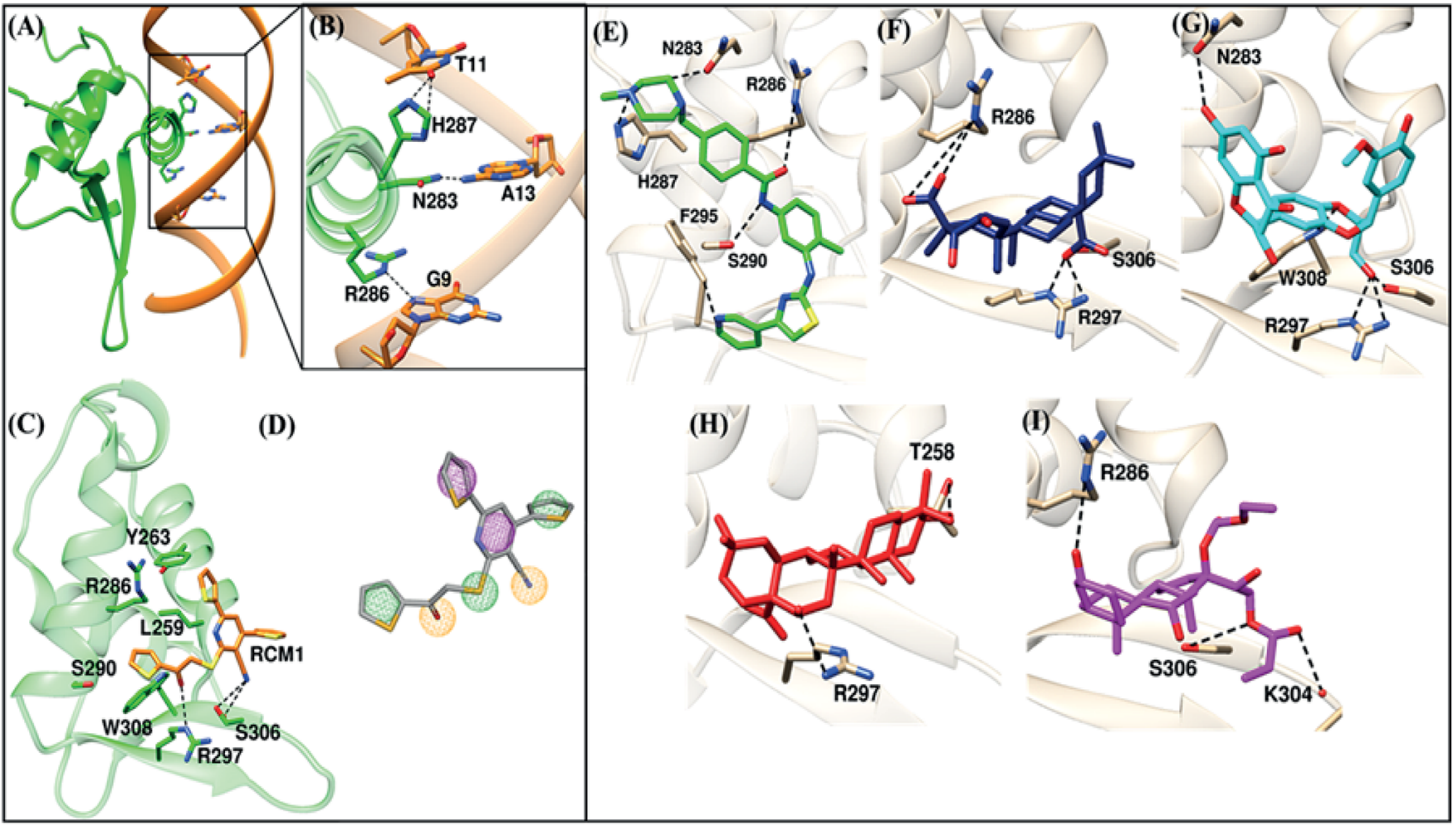
(A) Crystal structure of the FOXM1-DNA complex (PDB ID-3G73). (B) Zoomed representation depicting the binding interface of FOXM1-DBD (green sticks) and DNA (orange sticks) active site residues (green sticks). (C) FOXM1-RCM-1 (orange sticks) docked complex; interacting residues of FOXM1 are represented by green sticks, and hydrogen bonds are represented by black dashed lines. (D) Pharmacophore features of RCM-1 hydrophobic features are depicted by green spheres, hydrogen bond acceptor features are shown as orange spheres, and aromatic features are colored purple. Docked complex of the ligands within the active site of FOXM1 (E) Masitinib mesylate (F) Polygalic acid (G) Silibinin (H) Echinocystic acid (I) Prednicarbate. The interacting residues of FOXM1 are represented by tan sticks. Hydrogen bonds are represented by black dashed lines.

### 3.2 Pharmacophore-Based Virtual Screening

Pharmacophore modeling plays a crucial role in drug discovery and design, emerging as an essential tool in hit identification, lead optimization, and the rational design of new drugs based on the fundamental characteristics of a ligand. Using the Pharmit web server, two compound libraries from the Selleckchem database were screened with RCM-1 as a reference compound. Pharmacophore features were generated on the basis of the interaction pattern of the FOXM1-RCM-1 complex (Figure 1C); two were hydrogen bond acceptors, two were hydrophobic, and two were aromatic rings, as shown in Figure 1D. Based on pharmacophore features, 65 compounds from the FDA-approved anticancer library and 57 compounds from the natural product library from the Selleckchem database were sourced. Energy minimizations of all the screened compounds were performed in complex with FOXM1. These compounds subsequently underwent molecular docking with FOXM1 for interaction analysis.

### 3.3 Molecular Docking

Molecular docking is an effective method for screening potential compounds based on binding energy rankings and binding poses within the active site cavity of a receptor. The binding features of FOXM1 with DNA and RCM-1 were used to screen the 122 compounds and rerank them on the basis of the binding pose and binding energy of the reference compound (≥−6.4 kcal/mol), as shown in Figure 1C. By using the AutoDock protocol, 5 compounds with binding affinities equal to or greater than those of RCM-1 were screened. Based on the virtual screening results, five potential compounds were further selected for interaction analyses, the results of which are summarized in Figure 1E–I and Supporting Table 1. The screened compounds were further validated by molecular dynamics simulation by postdocking protein–ligand complexes (Table 1).

**Table 1:** Post-Docking Molecular Interactions of the Reference Compound (RCM-1) and the Top Selected Hits with Active Site Residues of FOXM1

compounds	binding energy (kcal/mol)	H-bonds	van der Waals interactions
RCM-1	–6.4	Arg 297, S306	Asn 283, His 287, Trp 308, Ser 290, Leu 259, Phe 307
Masitinib mesylate	–7.2	Asn 283, Arg 286, His 287, Ser 290, Phe 295	Trp 308, Leu 289, Thr 258, Ser306, Arg 297, Leu 259
Polygalic acid	–6.7	Arg 286, Arg 297, Ser 306	His 287, Ser 290, Trp 308, Thr 258
Silibinin	–6.9	Asn 283, Arg 297, Ser306, Trp 308	His 287, Lys 260, Tyr 263, Ser 290
Echinocystic acid	–6.6	Thr 258, Arg 297	His 287, Ser 290, Arg 297, Ser 306
Prednicarbate	–6.4	Arg286, Ser 306, Lys 304	Arg 297, Lys 282, Tyr 263, Val 305, Thr 258

### 3.4 ADMET Properties

Drug-likeness, pharmacokinetic, and toxicity investigations of the screened compounds and reference compounds were conducted to determine whether the compounds met the filter criteria (Supporting Table 2). AMES toxicity analysis indicated that all compounds are predicted to be nontoxic or have low toxicity, except silibinin, which shows greater AMES toxicity. Compared with RCM-1, three compounds, namely, masitinib mesylate, echinocystic acid, and prednicarbate, have good gastrointestinal absorption, and none of the compounds demonstrates permeability through the blood–brain barrier (BBB), suggesting a reduced impact on the central nervous system, as shown in the table. Additionally, when considering suitable properties such as lipophilicity (based on the iLOGP value), the appropriate range is between 0 and 5, and all studied compounds are within this range. The suitable range for TPSA is less than 140 Å^2^, and all studied compounds fall within this range except for silibinin, which indicates good oral bioavailability. Drug-likeness analysis indicated that all the compounds are suitable for drug synthesis (Table 2).

**Table 2:** ADMET Properties of the Compounds Selected through Molecular Docking

	compounds					
properties	RCM-1	Masitinib mesylate	polygalic acid	silibinin	echinocystic acid	prednicarbate
Hba	3	7	6	10	4	8
Hbd	0	5	4	5	4	5
AMES toxicity	0.7	0.2	0.1	0.9	0.3	0.2
TPSA	164	100	115	155	80	129
GIA	Low	High	Low	Low	High	High
BBB	No	No	No	No	No	No
Lipinski	Yes	Yes	Yes	Yes	Yes	Yes
Bioavailability score	0.55	0.55	0.56	0.55	0.55	0.55

### 3.5 Structural Stability and Trajectories of FOXM1-DBD Complexes during Simulation

To elucidate the conformational dynamics and stability of the screened compound complexes, MD simulations were conducted for 200 ns for each system in triplicate. All the screened compounds were stable in the active site cavity of the FOXM1 protein except silibinin. Ligand–protein complex stability was analyzed by calculating the root-mean-square deviation (RMSD) of the protein backbone atoms in relation to the equilibrated protein structure throughout the simulation. After docking, the effects of the compounds on the amino acid residues in the active site and the overall compactness of the system were analyzed by calculating the RMSF, *R*_g_, and SASA values (Supporting Table 3 and Table 3).

**Table 3:** Simulation Trajectories (RMSD, RMSF, *R*_g_ and SASA) of the FOXM1, FOXM1-RCM-1 and FOXM1-Inhibitor Complexes

complex	Avg. backbone RMSD (nm)	Avg. ligand RMSD (nm)	Avg. RMSF (nm)	Avg. SASA area (nm^2^)	Avg. *R*_g_ (nm)
FOXM1	0.166 ± 0.001	-	0.133 ± 0.004	64.950 ± 0.271	1.348 ± 0.011
FOXM1-RCM-1	0.164 ± 0.009	1.559 ± 0.611	0.158 ± 0.019	66.820 ± 0.521	1.367 ± 0.010
FOXM1-Masitinib mesylate	0.174 ± 0.027	0.994 ± 0.232	0.147 ± 0.026	68.123 ± 0.954	1.373 ± 0.010
FOXM1-Polygalic acid	0.151 ± 0.008	0.600 ± 0.274	0.166 ± 0.053	66.213 ± 0.882	1.359 ± 0.011
FOXM1-Silibinin	0.174 ± 0.023	2.475 ± 0.153	0.221 ± 0.034	66.997 ± 0.873	1.376 ± 0.017
FOXM1-Echinocystic acid	0.153 ± 0.001	0.949 ± 0.027	0.139 ± 0.006	66.843 ± 0.318	1.368 ± 0.002
FOXM1-Prednicarbate	0.170 ± 0.026	1.121 ± 0.115	0.170 ± 0.072	67.593 ± 2.028	1.381 ± 0.034

The protein backbone in complex with RCM-1 has a deviation of 0.166 nm. All four stable compounds show stable RMS deviation, ranging from 0.151 to 0.174 nm, reflecting the stability of the predicted compounds in the active site cavity of FOXM1-DBD (Figure 2A). The average ligand RMSDs in FOXM1 range from 0.600 to 2.475 nm; all four compounds deviate less from silibinin, which exhibited a large deviation of 2.475 nm compared with the reference RMSD of RCM-1 (1.559 nm) (Figure 2C), reflecting the compatibility of the predicted poses in the active sites of FOXM1. The root-mean-square fluctuations (RMSFs) of the backbone atoms were computed for each residue in all the systems and graphed over the course of the simulation. Residues on the DNA–protein interface (namely, Asn-283, Arg-286, His-287, Ser-290, and Arg-297) were generally stable, and their fluctuations were maintained during the simulation period. The average RMSF values for the ligand-bound and apo proteins were in the range of 0.133 to 0.221 nm, except for silibinin, which exhibited higher fluctuations than the reference compound did (Figure 2B). The solvent accessible surface area (SASA) and radius of gyration (*R*_g_) were also calculated to assess system equilibration. *R*_g_ quantifies the displacement of all atoms in a protein structure from a shared center of mass, offering insights into the overall compactness of the protein. On the other hand, SASA measures the exposed surface area of a protein structure and is related to protein–solvent interactions, providing information about protein conformation. The effect of ligand binding to the FOXM1 protein through *R*_g_ was studied and compared with that of the unbound protein. The compactness of the complexes, the FOXM1-RCM-1 complex, and apo FOXM1 were similar in the range of 1.348–1.381 nm (Figure 2D). The average SASA values of complexes were slightly increased, ranging from 64.950 to 68.123 (Figure 2E).

**Figure 2:**
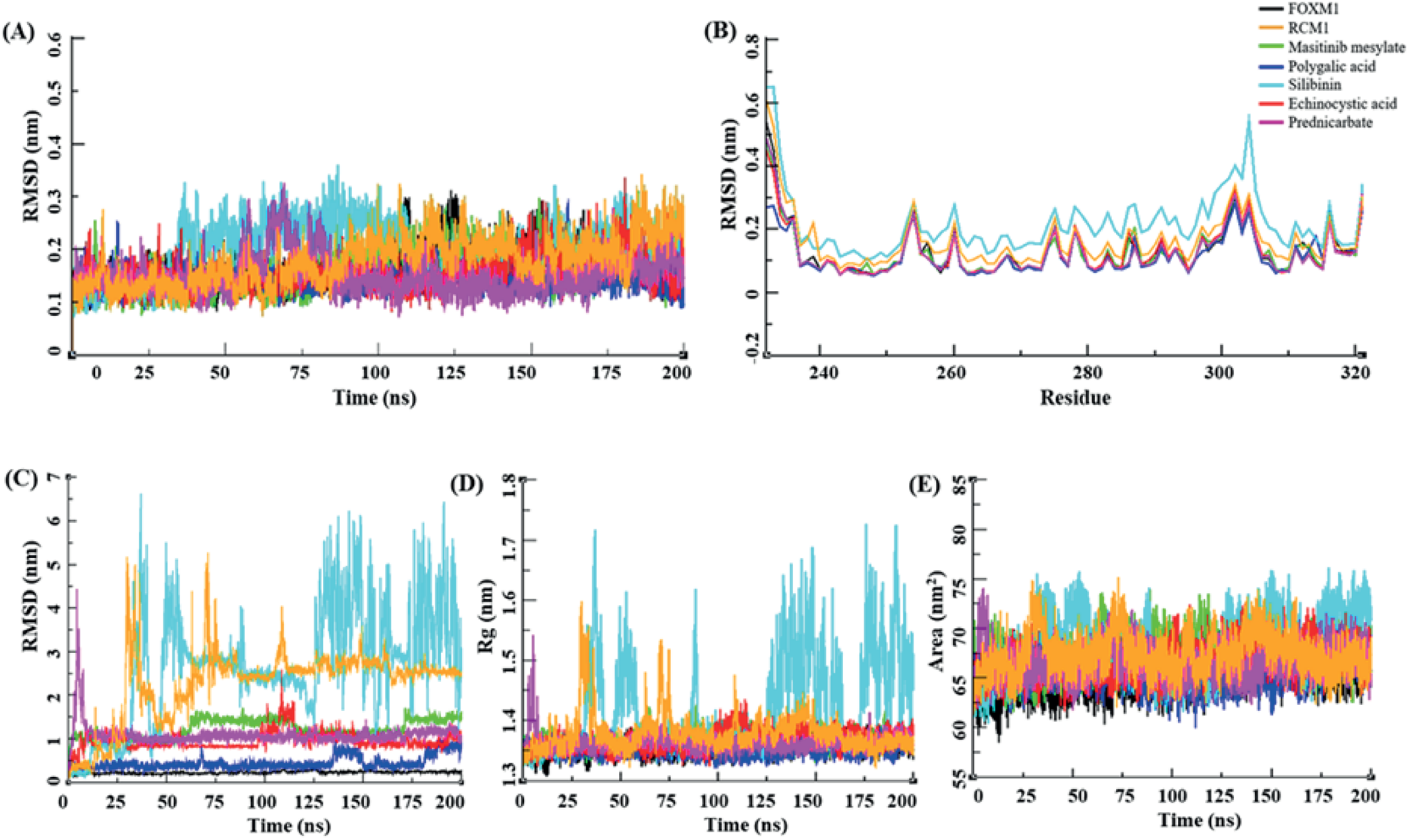
Trajectory analysis of FOXM1 and its complexes. (A) Root mean square deviation (RMSD, nm) of the protein backbone, (B) RMSF values of the α carbon over the entire simulation, (C) ligand root-mean-square deviation (RMSD, nm), (D) radius of gyration (*R*_g_), and (E) solvent accessible surface area (SASA) over the entire simulation. FOXM1 is represented in black, FOXM1-RCM-1 in orange, FOXM1-Masitinib mesylate in green, FOXM1-Polygalic acid in blue, FOXM1-Silibinin in cyan, FOXM1-Echinocystic acid in red, and FOXM1-Prednicarbate in purple.

### 3.6 Postsimulation Protein–Ligand Interaction Analysis

For an inhibitor to be considered a viable drug candidate, it must bind effectively to the active site cavity with strong affinity. As discussed in the previous section, with the exception of silibinin, all four compounds remained stable within the binding pocket of the FOXM1-DNA interface. The binding patterns of these four compounds were analyzed *via* post simulation protein–ligand complexes, and the results are summarized in Figure 3. Masitinib mesylate interacts with FOXM1 by making two hydrogen bonds with residues His 287 and Leu 291, a salt bridge with Asp293, and three van der Waals interactions with residues Gly 280, Ser 284 and Ser 290. Polygalic acid forms two hydrogen bonds with residues Arg 286 and Ser 308; two salt bridges with residues Arg 286 and Arg 297; and van der Waals interactions with Ser290, Leu289, and Leu259. Similarly, prednicarbate forms two hydrogen bonds with Arg286 and one with Arg297 and five van der Waals interactions with the key active site residues Asn283, His287, Leu289, Ser290, and Trp308. Echinocystic acid forms one H-bond and one salt bridge with residue Arg 297 and two van der Waals interactions involving Thr299 and Ser306.

**Figure 3:**
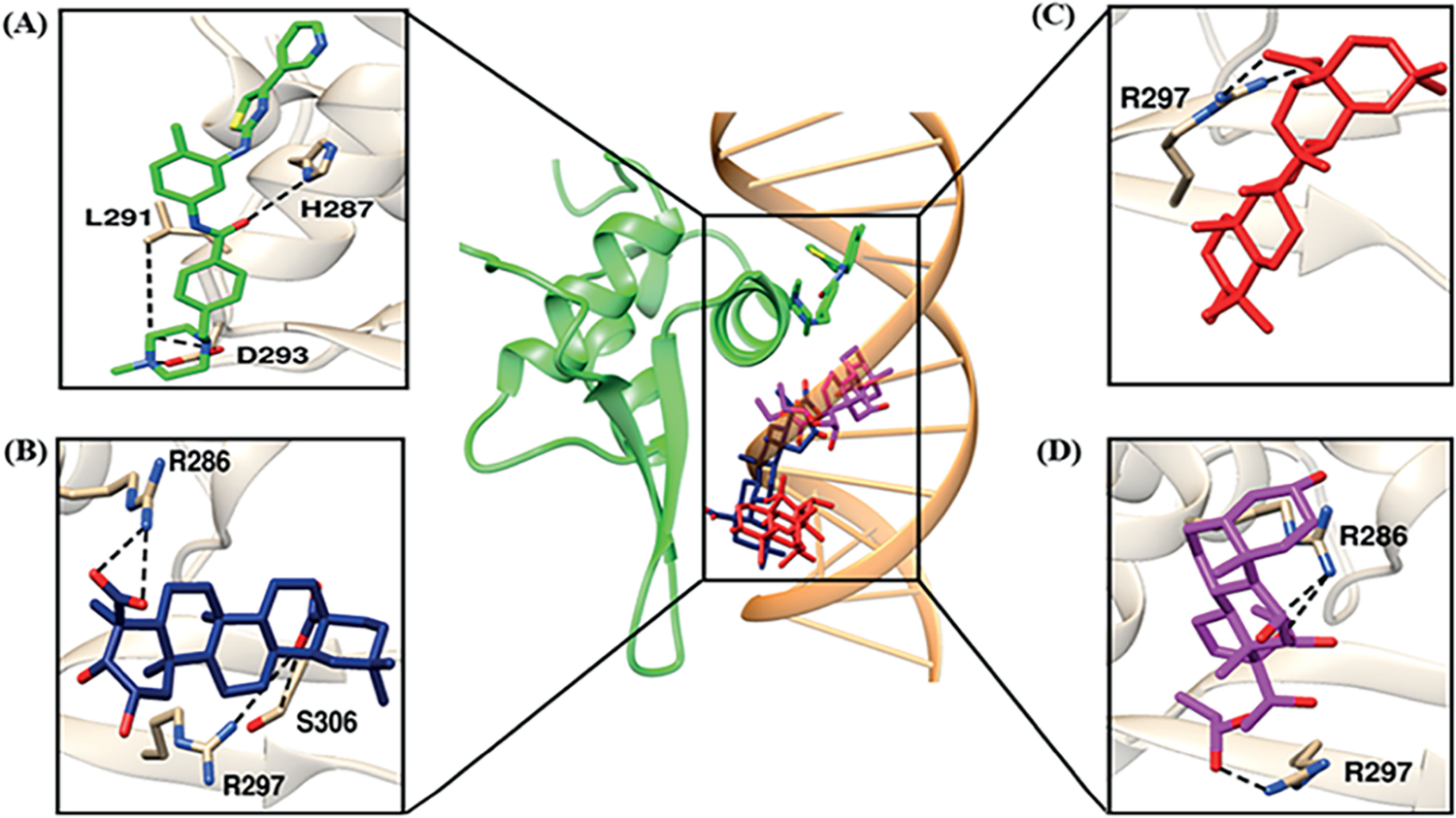
Postsimulation binding pattern of the ligands within the active sites of FOXM1 (A) masitinib mesylate (B) polygalic acid (C) echinocystic acid (D) prednicarbate. The interacting residues of FOXM1 are represented by tan sticks. Hydrogen bonds are represented by dashed black lines.

### 3.7 Masitinib Inhibits the Viability, Proliferation, and Colony Formation of Cancer Cell Lines and *Ex Vivo* Tissue Slice Culture

Treatment with masitinib markedly inhibited the cell viability, proliferation, and clonogenic potential of breast cancer cells MDA-MB-231 and HNSCC cells HSC-3 in a dose- and time-dependent manner. MTT cell viability assays were used to determine the IC_50_ values of masitinib mesylate in both cell lines. HSC-3 cells demonstrated an IC_50_ of 1.5 μM (95% CI: 1.1–4.9 μM), indicating high sensitivity to FOXM1 inhibition. MDA-MB-231 cells presented an IC_50_ of 10 μM (95% CI: 6.2–12.2 μM), indicating sensitivity to treatment. As shown in Figure 4A, the percentage of viable cells decreased progressively with increasing masitinib concentration, indicating significant cytotoxicity in both models. Time-course analyses (Figure 4B) revealed pronounced suppression of cell growth, with masitinib-treated cells exhibiting reduced proliferation and viability over 24–72 h compared to untreated control cells. Furthermore, clonogenic assays (Figure 4C) confirmed that masitinib disrupted long-term cell survival, with the number of colonies formed by both cell lines decreasing sharply with increasing drug concentrations and reaching near-complete inhibition at the highest doses. Together, these findings demonstrate that masitinib exerts potent antiproliferative and cytotoxic effects on these cancer cells, significantly reducing viability, proliferation, and colony formation in a concentration- and time-dependent fashion. Compared with the control cells, the proliferation rates of both cell lines decreased when treated with the drug at the IC_50_ for up to 72 h. Compared with control cells, colony formation assays revealed fewer and smaller colonies in masitinib-treated cells, suggesting a significant reduction in tumor growth rate.

**Figure 4:**
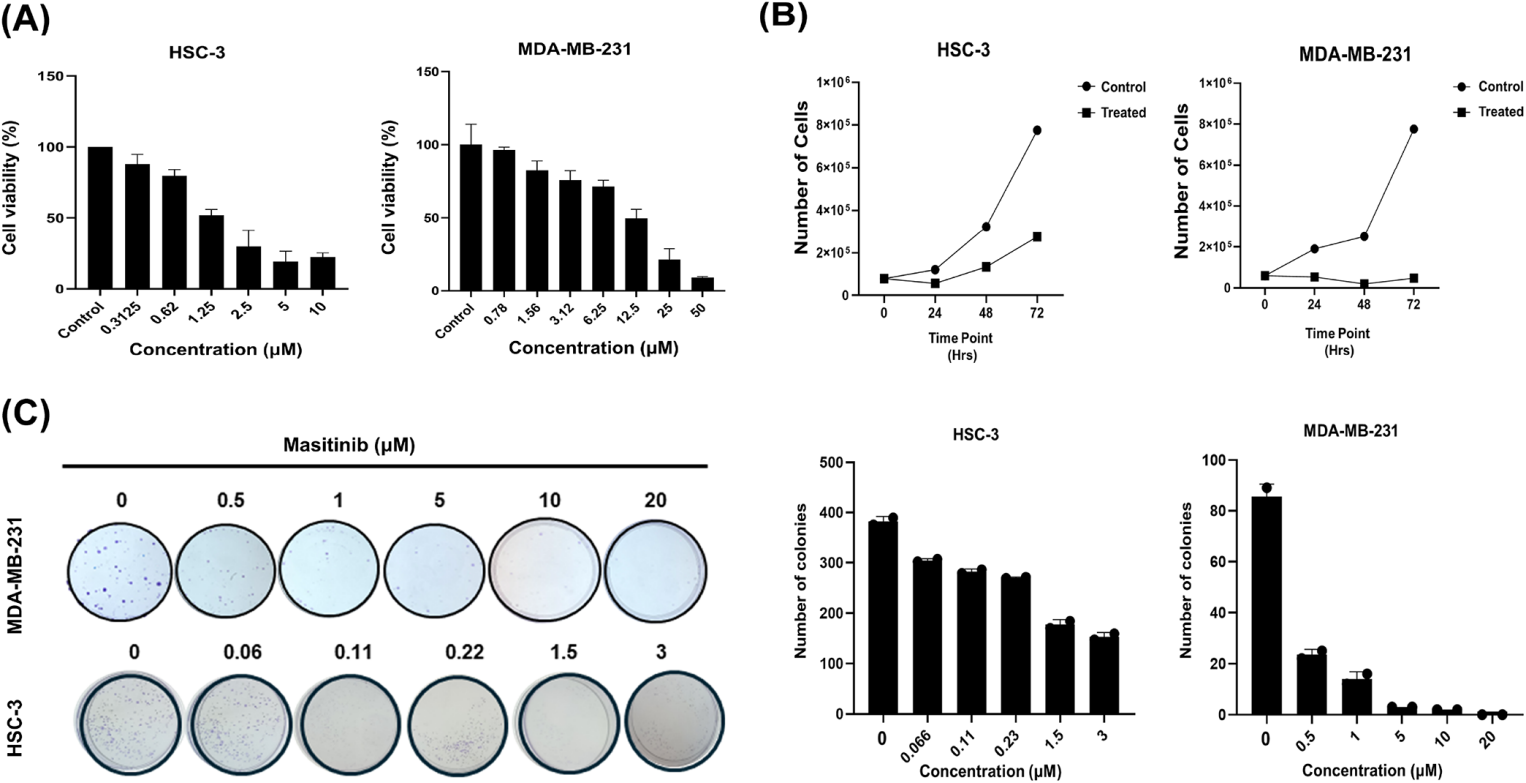
Masitinib mesylate inhibits the viability and colony formation of cancer cells. (A) Dose–response curves showing the effects of masitinib mesylate on the HSC-3 and MDA-MB-231 cell lines. The cells were treated with increasing concentrations of each compound, and viability was assessed *via* a standard cell viability assay. The data are presented as the means ± SDs (*n* = 3). (B) Cell proliferation assay of the MDA-MB-231 and HSC-3 cell lines treated with the test compound compared with the control over 72 h. Cell counts were performed at 0, 24, 48, and 72 h. The error bars indicate the SDs from triplicate experiments. (C) Colony formation assay showing the effect of masitinib on cell proliferation. Representative images of stained colonies are shown, along with colony counts. The data are presented as the means ± SDs, with statistical significance indicated (**p < 0.01, ***p < 0.001).

To evaluate masitinib’s selectivity, we assessed its cytotoxicity in the noncancerous human keratinocyte cell line (HaCaT) and human embryonic kidney (HEK293) cells. Importantly, masitinib did not show cytotoxicity across the tested concentration range (1.25–20 μM) in breast and HNSCC cells (Supporting Figure S1), indicating selective activity against cancer cells. These findings diminish the likelihood that the observed FOXM1 inhibition results from nonspecific cytotoxic stress.

### 3.8 Masitinib Downregulates FOXM1 Expression and Cell Cycle Progression in Cancer Cells

To evaluate the effects of masitinib on FOXM1 protein expression and function, immunoblot analysis was performed. We observed a significant reduction in FOXM1 protein expression in breast cancer and HNSCC cell lines following masitinib treatment, compared with untreated controls at their respective IC_50_ concentrations (Figure 5A). Thiostrepton, a known FOXM1 inhibitor, was used as a positive control and showed a similar reduction in FOXM1 expression in breast cancer and HNSCC cells (Figure 5A). Although we observed multiple FOXM1-immunoreactive bands consistent with previously reported post-translational modifications and isoforms, masitinib treatment consistently reduced FOXM1 expression in both HNSCC and breast cancer cell lines. To further investigate whether FOXM1 inhibition by masitinib perturbs cell cycle progression, we assessed Cyclin B1 (CCNB1) expression, a well-established FOXM1 transcriptional target involved in G2/M cell cycle progression. Masitinib treatment resulted in a marked reduction in Cyclin B1 expression in both breast and HNSCC cell lines, suggesting perturbation in FOXM1 regulation of the cell cycle in cancer cells (Supporting Figure S2).

**Figure 5:**
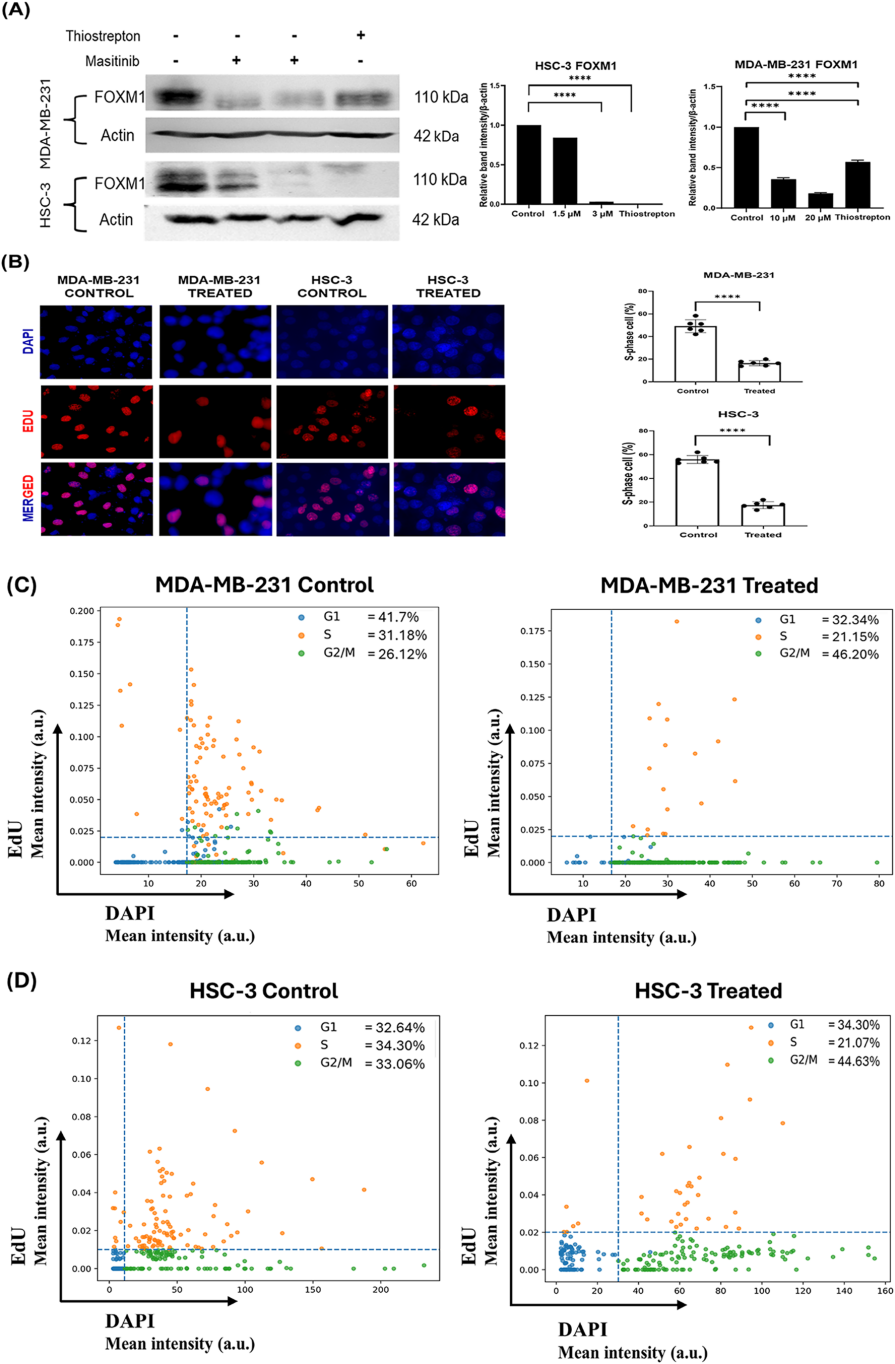
Masitinib mesylate inhibits FOXM1 expression and cell proliferation in cancer cells. (A) Western blot analysis showing FOXM1 protein levels in HSC-3 cells treated with masitinib (1.5 μM and 3 μM) and MDA-MB-231 with masitinib (10 μM and 20 μM) or Thiostrepton (1 μM). β-actin was used as a loading control. Representative blots are shown along with densitometric quantification of relative band intensities normalized to β-actin. The values are expressed relative to the control values and represent the means ± SEMs from three independent experiments. Statistical significance was determined *via* one-way ANOVA (*p < 0.05, ** *p* < 0.01, ***p < 0.001, ****p < 0.0001). (B) Representative fluorescence microscopy images of the EdU incorporation assay in control and masitinib-treated MDA-MB-231 and HSC-3 cells. Nuclei were stained with DAPI (blue), and EdU-positive cells (red) indicated actively replicating DNA. Merged images showing the colocalization of EdU and DAPI. The percentage of S-phase cells is shown in the bar graphs. Statistical significance was assessed by an unpaired Student’s *t* test (****p < 0.0001). (C and D) Representative QIBC scatter plots showing DAPI integrated intensity*versus* EdU incorporation in control and treated cells. Nuclei were segmented using DAPI staining, and EdU incorporation was quantified per nucleus. Cells were classified into G1, S, and G2/M phases based on DNA content and EdU incorporation.

EdU incorporation was assessed to specifically evaluate effects on cell proliferation and analyze S-phase progression in cancer cells. Imaging analysis revealed significant reductions in the number of EdU-positive S-phase cells in both breast cancer and HNSCC cell lines. In MDA-MB-231 cells treated with the IC_50_ concentration (10 μM), the percentage of EdU-positive cells decreased from 52.3% in the control cells to 18.5% in the treated cells, representing a 56% reduction in proliferating cells (Figure 5B). Similarly, the number of EdU-positive S-phase cells in the HSC-3 cell line treated with the IC_50_ concentration (1.5 μM) decreased from 58% in the control group to 16% following treatment, representing a 55% reduction (Figure 5B). To investigate whether masitinib mediated FOXM1 inhibition perturb cell-cycle progression, QIBC analysis was performed using EdU incorporation and DAPI intensity measurements. Single-cell analysis enabled classification of cells into G1, S, and G2/M populations based on DNA synthesis activity and overall DNA content. Masitinib-treated cells showed decreased S-phase cell population (control, 31.18% *vs* masitinib-treated, 21.15%) in breast cancer cells and (control, 34.30% *vs* masitinib-treated, 21.07%) in HNSCC cells (Figure 5C–D). Further, we observed an increase in G2/M phase cells (control, 26.12% *vs* masitinib-treated, 46.20%) in breast cancer cells and (control, 33.06% *vs* masitinib-treated, 44.63%) in HNSCC cells, suggesting G2/M arrest (Figure 5C–D). This observation supported the downregulation of the FOXM1 target gene, CCNB1, in masitinib-treated cells (Supporting Figure S1), which is crucial for the G2/M transition.

### 3.9 Masitinib Promote Cell Death in Organotypic Tumor Slice Culture

To evaluate the clinical relevance of masitinib, primary breast cancer patient-derived tumor tissue slices (*n* = 3) and HNSCC patient-derived tumor tissue slices (*n* = 3) were incubated with or without the drug for 72 h. Details of the clinical samples are provided in Supporting Table 4 and Table 4.

**Table 4:** Clinical and Pathological Features of Breast and Head and Neck Carcinomas Included in the *Ex Vivo* Drug Response Study

sample ID	tumor type	primary site	age (Y)	sex	stage	grade	*ex vivo* response
BC-01	Breast	Lower quadrant	58	F	pT2N0M0	II	↓EdU, ↑TUNEL
BC-02	Breast	Upper Inner Quadrant	37	F	pT1cN1aM0	I	↓EdU, ↑TUNEL
BC-03	Breast	Lower outer quadrant	37	F	pT3N2M	III	↓EdU, ↑TUNEL
HN-01	HNSCC	Posterior pharyngeal	64	M	pT2N3bM0 (IV B)	I	↓EdU, ↑TUNEL
HN-02	HNSCC	Lower GBS	57	M	pT3N1M0 (III)	III	↓EdU, ↑TUNEL
HN-03	HNSCC	Oral tongue	49	F	pT1N0M0 (I)	II	↓EdU, ↑TUNEL

Histological analysis using H&E staining revealed alterations in tumor tissue architecture indicative of cell shrinkage and nuclear condensation in masitinib-treated breast and HNSCC tumor slices compared to untreated tumor slices (Figure 6A,6D). Furthermore, quantitative imaging analysis of EdU incorporation demonstrated a marked reduction in S-phase cells and a significant increase in TUNEL-positive apoptotic cells in masitinib-treated HNSCC and breast tumor slices compared with untreated control tumor slices (Figure 6A–E, and Supporting Figure S3). These data further validate the clinical efficacy of masitinib, which inhibits tumor cell proliferation (decreased S-phase cells) and enhances apoptosis (increased TUNEL-positive cells) in two distinct organotypic tumor slice culture models.

**Figure 6:**
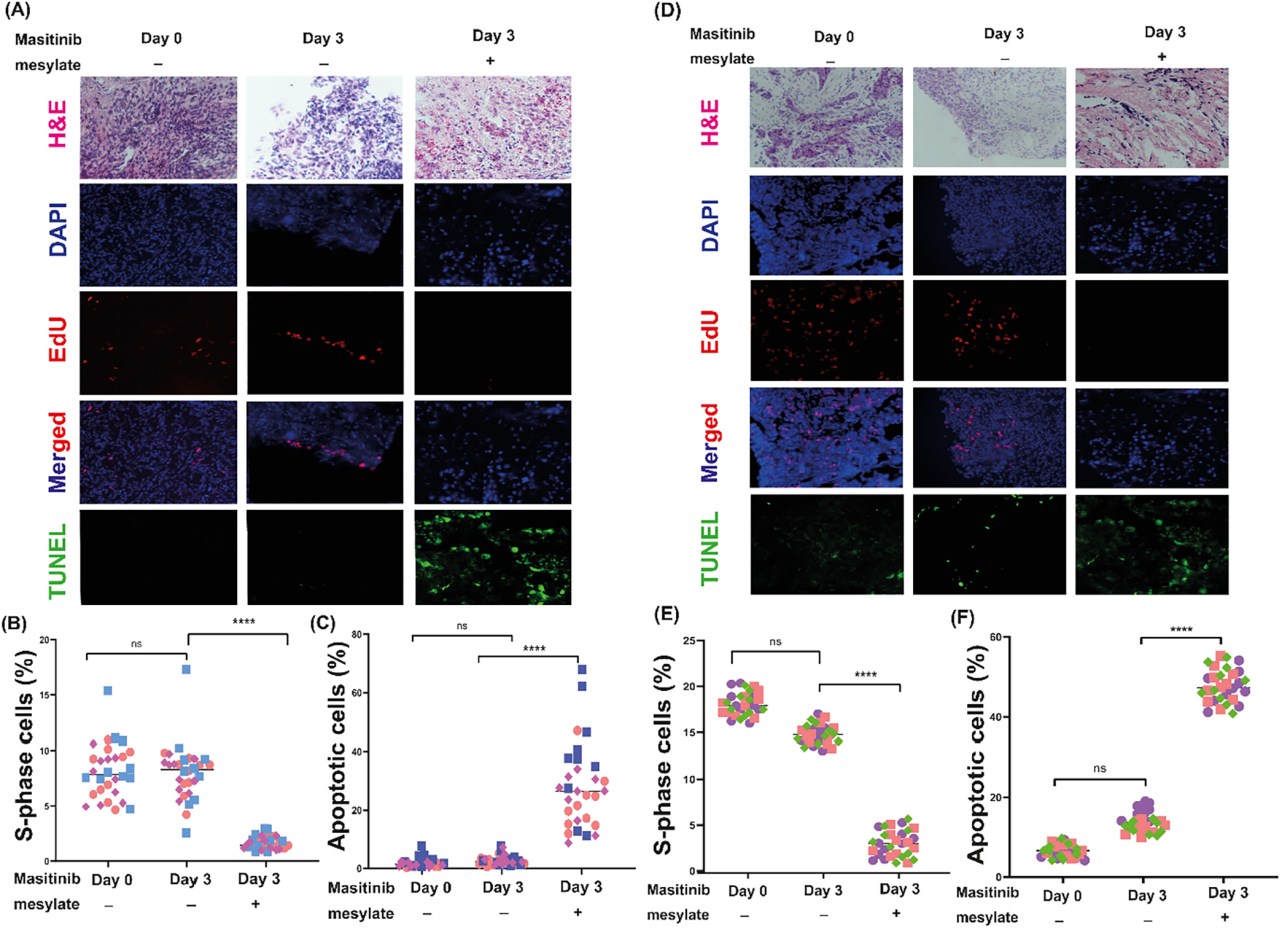
Masitinib mesylate suppresses proliferation and induces apoptosis in HNSCC cancer and breast cancer tumor slice cultures. (A) Representative images of hematoxylin and eosin (H&E) staining, DAPI, EdU, merged DAPI/EdU, and TUNEL staining of the tumor. (B) Quantification of proliferating S-phase cells, expressed as the percentage of EdU-positive cells per total DAPI-positive nuclei for each slice; data from individual tumors are shown as symbols with mean indicated (ns, not significant; ****, *P* < 0.0001). (C) Quantification of apoptotic cells, expressed as the percentage of TUNEL-positive cells per total DAPI-positive nuclei; symbols represent individual tumors with mean indicated (ns, not significant, *P* < 0.0001). (D) Representative images of hematoxylin and eosin (H&E) staining, DAPI, EdU, merged DAPI/EdU, and TUNEL staining of the tumor. (E) Quantification of proliferating S-phase cells, expressed as the percentage of EdU-positive cells per total DAPI-positive nuclei for each slice; data from individual tumors are shown as symbols with mean indicated (ns, not significant; ****, *P* < 0.0001). (F) Quantification of apoptotic cells, expressed as the percentage of TUNEL-positive cells per total DAPI-positive nuclei; symbols represent individual tumors with mean indicated (ns, not significant, *P* < 0.0001). Data points represent 10 fields per sample for 3 independent tumor samples (*n* = 3) for both EdU and TUNEL staining.

## 4 Discussion

Over the past 50 years, cancer research has yielded a substantial body of knowledge; however, advancements in therapeutic strategies for patients with solid tumors have been limited, resulting in only a modest increase in patient survival, typically extending life by mere months. Solid tumors, including breast cancer (BC) and head and neck squamous cell carcinoma (HNSCC), are notably associated with poor survival outcomes, particularly in patients with triple-negative breast cancer (TNBC) and advanced-stage metastatic HNSCC. Consequently, this study employs the MDA-MB-231 (TNBC subtype) and HSC-3 (metastatic HNSCC) cell lines. The forkhead family of transcription factors (FOXM1) is recognized as a critical regulator of cell proliferation and mitosis, influencing the transcription of numerous genes and exhibiting overexpression in various cancers, such as BC and HNSCC. The extensive and diverse role of FOXM1 in carcinogenesis and therapy resistance across different tissues positions it as a potentially promising target for inhibition in anticancer treatment.

The present study elucidates the function of the small-molecule inhibitor masitinib mesylate in suppressing FOXM1 activity within the MDA-MB-231 and HSC-3 cell lines. Previously, RCM-1, a small-molecule inhibitor, impairs FOXM1 by blocking its nuclear localization and enhancing its ubiquitination, thereby inhibiting tumor growth, reducing cell proliferation, and increasing apoptosis in cell lines such as B16–F10 and H2122, as well as in murine models, therefore we used RCM-1 as the reference small molecule inhibitor for the identification of masitinib mesylate as potent inhibitor of FOXM1.^24,38^

Over the past decade, computer-aided drug design and discovery methods have been highly effective in developing new small molecules from known compounds and repurposing drugs for similar functions. We used RCM-1 as the reference molecule to identify natural lead small-molecule inhibitors in our study. This study focused primarily on the small-molecule inhibitor masitinib mesylate. In veterinary medicine, masitinib is used to treat recurrent or unresectable canine mast tumors. Moreover, Masitinib primarily targets the KIT and PDGFR family kinases, which are recognized as upstream regulators of the MAPK/ERK signaling pathways.^39^ The activity and stability of FOXM1 are significantly influenced by MAPK/ERK-mediated phosphorylation and nuclear translocation. Consequently, the decrease in FOXM1 expression observed after Masitinib treatment might also be indirectly due to the inhibition of upstream MAPK/ERK signaling pathways.^39^ Regulation of the cell cycle by FOXM1 is predominantly facilitated through its phosphorylation and subsequent activation, which are crucial for its function during the G2/M phase. Our observations indicate that treatment with masitinib in breast cancer and head and neck squamous cell carcinoma (HNSCC) cells reduced CCNB1 expression, a recognized FOXM1 target gene essential for the G2/M transition. Quantitative image-based cytometry (QIBC) analysis corroborated G2/M arrest in both breast cancer and HNSCC cell lines, thereby substantiating masitinib’s role in inducing cell death in these cell lines and in organotypic tumor models.

## 5 Conclusion

We demonstrate that masitinib suppresses FOXM1 expression and activity, thereby reducing proliferation and increasing apoptosis in cancer models. While our findings support FOXM1 functional inhibition, further studies are needed to distinguish between direct target engagement and indirect regulatory mechanisms. Furthermore, *ex vivo* assessment of masitinib response was performed using an organotypic tumor tissue slice platform with primary breast cancer (*n* = 3) and head and neck cancer (*n* = 3). By analyzing primary tumor tissue architecture, cell viability, and apoptosis, masitinib treatment consistently showed decreased proliferation and increased apoptosis in primary breast cancer and HNSCC samples, indicating robust drug-induced cytotoxicity in both tumor types. These findings support further preclinical and clinical evaluation of masitinib-based strategies in breast cancer and HNSCC. Future studies will explore the therapeutic role of masitinib in both *in vivo* and *ex vivo* settings in combination with radiation and platinum-based chemotherapy drugs for both head and neck cancer and breast cancer. While our findings support masitinib’s inhibition of FOXM1 activity, further studies using genetic approaches, such as a FOXM1 knockout model, are needed to definitively assess target specificity and potential off-target effects.

## Supplementary Material



## Data Availability

All data sets generated for this study are included in the article/Supporting Information.

## Notes

Detailed molecular docking interactions of RCM-1 and the top five screened compounds with the FOXM1 DNA-binding domain, including binding energies and interacting amino acid residues (Supporting Table S1); comprehensive ADMET profiling of the selected compounds, including physicochemical properties, toxicity prediction, gastrointestinal absorption, blood-brain barrier permeability, and drug-likeness parameters (Supporting Table S2); summary of molecular dynamics simulation parameters (RMSD, RMSF, radius of gyration, and SASA) for apo FOXM1 and FOXM1-ligand complexes (Supporting Table S3); clinicopathological characteristics of breast cancer and HNSCC patient samples used for the *ex vivo* tumor slice culture experiments (Supporting Table S4); effect of masitinib on the viability of nonmalignant HEK-293 and HaCaT cells, demonstrating minimal cytotoxicity at the concentrations used in this study (Supporting Figure S1); western blot validation showing reduced expression of the FOXM1 downstream target Cyclin B1 (CCNB1) following masitinib treatment, together with densitometric quantification (Supporting Figure S2); higher-magnification images of *ex vivo* tumor slice cultures highlighting reduced EdU incorporation and increased TUNEL staining in masitinib-treated breast cancer and HNSCC tissues (Supporting Figure S3) (PDF)
