# Supplementary material for: Repurposing Masitinib Mesylate as a Novel FOXM1 Inhibitor for the Treatment of Aggressive Solid Tumors: Preclinical Validation in Human Breast and Oral Cancer Cells and Organotypic Tumor Slice Culture
Source: ACS Omega. 2026 Jun 29;11(27):40323–35. doi: 10.1021/acsomega.6c02787 (PMC13382680; doi:10.1021/acsomega.6c02787)
Supplement: Supplementary file 1 [file ao6c02787_si_001.pdf]

Repurposing masitinib mesylate as a novel FOXM1 inhibitor for the treatment of aggressive solid tumours: preclinical validation in human breast and oral cancer cells and organotypic tumour slice culture

Rajat Gupta[a], Jai Singh[b], Shruti Dayanand Shetty[a], Naveena A N Kumar[d], Adarsh Kudva[e], Sandeep Kumar Srivastava[b,c]*, Sanjiban Chakrabarty[a]*

[a]Department of Public Health Genomics, Manipal School of Life Sciences, Manipal Academy of Higher Education, Manipal, India.

[b]Structural Biology & Bioinformatics Laboratory, Department of Biosciences, Manipal University Jaipur, Jaipur 303007, Rajasthan, India.

[c]Department of Biochemistry, University of Allahabad, Prayagraj 211002, Uttar Pradesh, India

[d]Department of Surgical Oncology, Manipal Comprehensive Cancer Care Centre, Kasturba Medical College, Manipal Academy of Higher Education, Manipal 576104, Karnataka, India.

[e]Department of Oral and Maxillofacial Surgery, Manipal College of Dental Sciences, Manipal, Manipal Academy of Higher Education, Manipal 576104, Karnataka, India.

***Corresponding author**

sandeepks@allduniv.ac.in

sanjiban.c@manipal.edu

Supplementary Data

Supplementary Tables

Supplementary Table 1: Post-docking molecular interactions of the reference compound (RCM-1) and the top selected hits with active site residues of FOXM1.

**Compounds**	**Binding energy (Kcal/mol)**	**H-bonds**	**van der Waals interactions**
**RCM-1**	-6.4	Arg 297, S306	Asn 283, His 287, Trp 308, Ser 290, Leu 259, Phe 307
**Masitinib mesylate**	-7.2	Asn 283, Arg 286, His 287, Ser 290, Phe 295	Trp 308, Leu 289, Thr 258, Ser306, Arg 297, Leu 259

**Polygalic acid**	-6.7	Arg 286, Arg 297, Ser 306	His 287, Ser 290, Trp 308, Thr 258
**Silibinin**	-6.9	Asn 283, Arg 297, Ser306, Trp 308	His 287, Lys 260, Tyr 263, Ser 290
**Echinocystic acid**	-6.6	Thr 258, Arg 297	His 287, Ser 290, Arg 297, Ser 306
**Prednicarbate**	-6.4	Arg286, Ser 306, Lys 304	Arg 297, Lys 282, Tyr 263, Val 305, Thr 258

Supplementary Table 2: ADMET properties of the compounds selected through molecular docking.

**Properties**	**Compounds**					
	**RCM-1**	**Masitinib mesylate**	**Polygalic acid**	**Silibinin**	**Echinocystic acid**	**Prednicarbate**
**Hba**	3	7	6	10	4	8
**Hbd**	0	5	4	5	4	5
**AMES toxicity**	0.7	0.2	0.1	0.9	0.3	0.2
**TPSA**	164	100	115	155	80	129
**GIA**	Low	High	Low	Low	High	High
**BBB**	No	No	No	No	No	No
**Lipinski**	Yes	Yes	Yes	Yes	Yes	Yes
**Bioavailability score**	0.55	0.55	0.56	0.55	0.55	0.55

Supplementary Table 3: Simulation trajectories (RMSD, RMSF, R_g and SASA) of the FOXM1, FOXM1-RCM-1 and FOXM1-inhibitor complexes.

**Complex**	**Avg. backbone RMSD (nm)**	**Avg. ligand RMSD (nm)**	**Avg. RMSF(nm)**	**Avg. SASA area(nm^2)**	**Avg. Rg(nm)**
**FOXM1**	0.166 ± 0.001	--	0.133 ± 0.004	64.950 ± 0.271	1.348 ± 0.011
**FOXM1-RCM-1**	0.164 ± 0.009	1.559 ± 0.611	0.158 ± 0.019	66.820 ± 0.521	1.367 ± 0.010
**FOXM1-Masitinib mesylate**	0.174 ± 0.027	0.994 ± 0.232	0.147 ± 0.026	68.123 ± 0.954	1.373 ± 0.010
**FOXM1-Polygalic acid**	0.151 ± 0.008	0.600 ± 0.274	0.166 ± 0.053	66.213 ± 0.882	1.359 ± 0.011
**FOXM1-Silibinin**	0.174 ± 0.023	2.475 ± 0.153	0.221 ± 0.034	66.997 ± 0.873	1.376 ± 0.017
**FOXM1-Echinocystic acid**	0.153 ± 0.001	0.949 ± 0.027	0.139 ± 0.006	66.843 ± 0.318	1.368 ± 0.002
**FOXM1-Prednicarbate**	0.170 ± 0.026	1.121 ± 0.115	0.170 ± 0.072	67.593 ± 2.028	1.381 ± 0.034

Supplementary Table 4: Clinical and pathological features of breast and head and neck carcinomas included in the ex vivo drug response study.

Sample ID	Tumor type	Primary site	Age (Y)	Sex	Stage	Grade	ex vivo response
**BC-01**	Breast	Lower quadrant	58	F	pT2N0M0	II	↓EdU, ↑TUNEL
**BC-02**	Breast	Upper Inner Quadrant	37	F	pT1cN1aM0	I	↓EdU, ↑TUNEL
**BC-03**	Breast	Lower outer quadrant	37	F	pT3N2M	III	↓EdU, ↑TUNEL
**HN-01**	HNSCC	Posterior pharyngeal	64	M	pT2N3bM0 (IV B)	I	↓EdU, ↑TUNEL
**HN-02**	HNSCC	Lower GBS	57	M	pT3N1M0 (III)	III	↓EdU, ↑TUNEL
**HN-03**	HNSCC	Oral tongue	49	F	pT1N0M0 (I)	II	↓EdU, ↑TUNEL

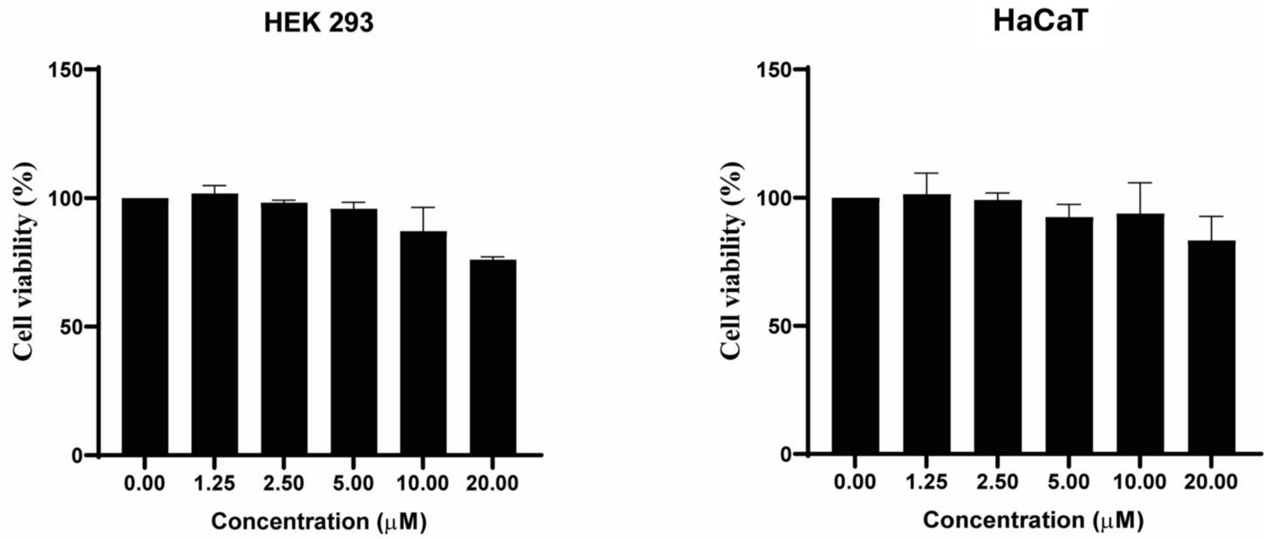

Supplementary Fig. S1: Effect of masitinib on cell viability assessed by MTT assay. Cell viability of **S2(A)** HEK-293 and **(B)** HaCaT cells following treatment with increasing concentrations of masitinib for 72 h was measured using the MTT assay. Viability is expressed as a percentage relative to the untreated control. Data represent mean ± SD of three independent experiments. Statistical significance was determined using [Student's t-test] (ns = not significant).

(A)

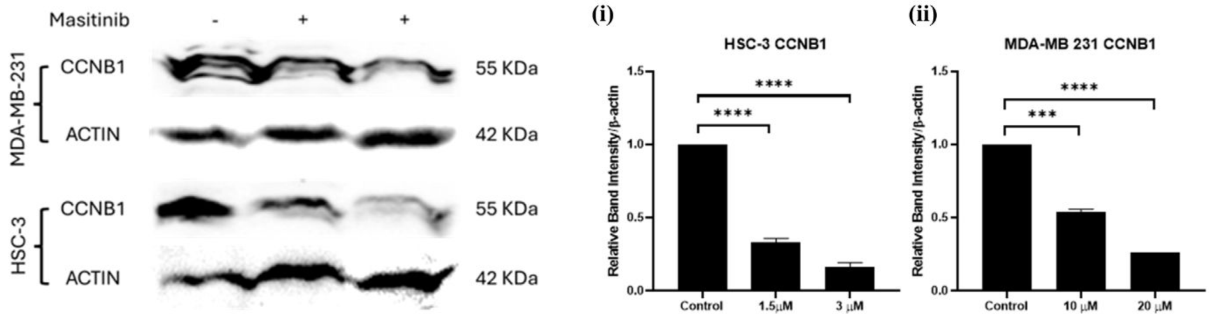

Supplementary Fig. S2: Masitinib suppresses FOXM1 target gene Cyclin B1 (CCNB1). (A) Western blot analysis showing Cyclin B1 expression in HNSCC & Breast cancer cells following treatment with masitinib at the indicated concentrations. A reduction in Cyclin B1 levels was observed upon treatment, consistent with FOXM1 transcriptional activity inhibition. β-actin was used as a loading control. **S1(i-ii)** Densitometric quantification represents relative protein levels normalised to β-actin (***$p < 0.001$, ****$p < 0.0001$).

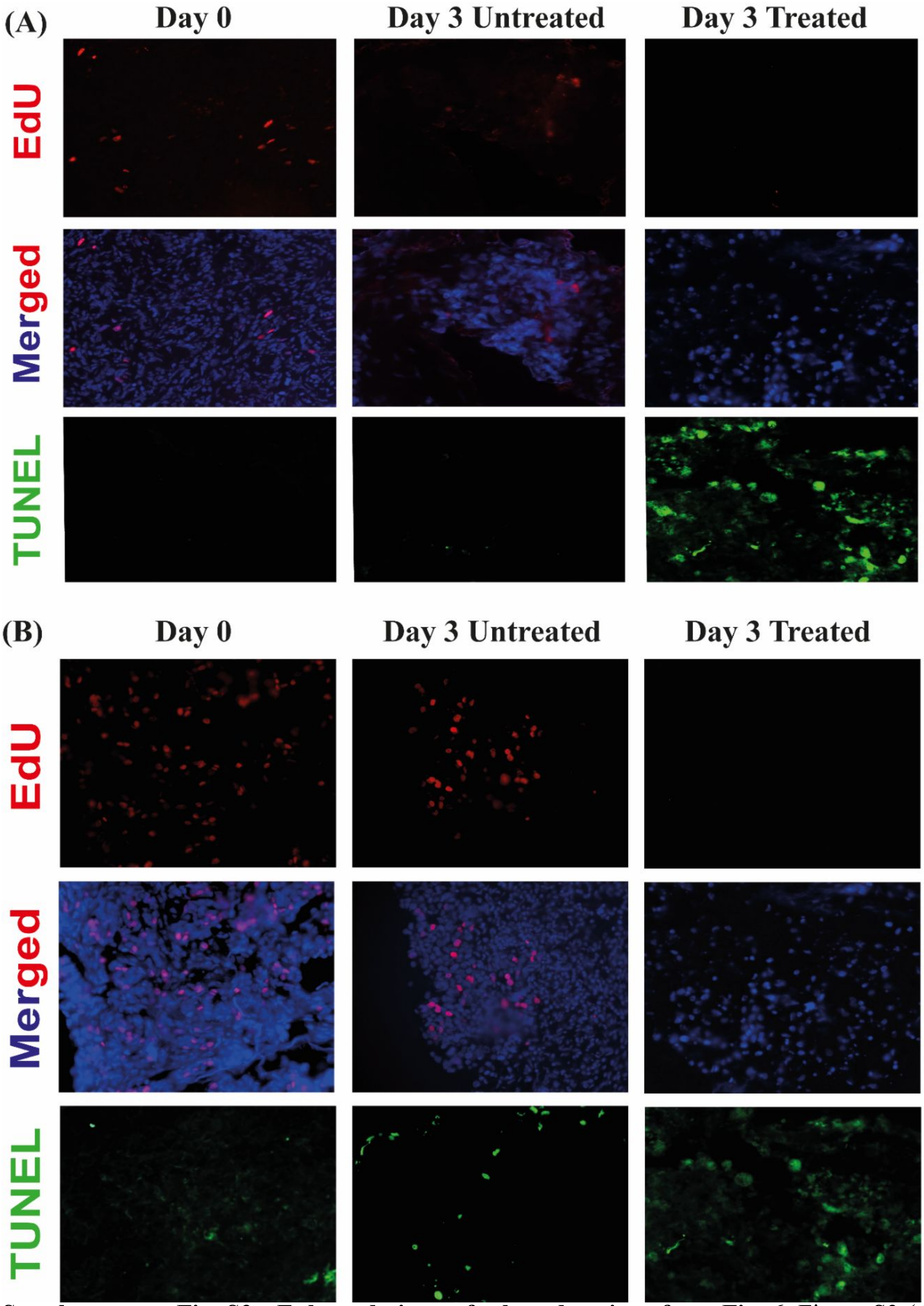

Supplementary Fig. S3: Enlarged views of selected regions from Fig. 6. Figure S3 (A) is the enlarged view of the head and neck patient tissue samples for the EdU, merged EdU-DAPI and TUNEL. Figure S3 (B) is the enlarged view of the breast cancer tissue samples for the EdU, merged EdU-DAPI and TUNEL.
